# Canadian dairy farmer views about animal welfare

**DOI:** 10.1017/awf.2023.32

**Published:** 2023-05-15

**Authors:** Catherine A Schuppli, Jeffrey M Spooner, Marina AG von Keyserlingk

**Affiliations:** The University of British Columbia, Animal Welfare Program, British Columbia, Canada

**Keywords:** Animal welfare, cows, dairy production, farmer attitudes, human-animal interaction, qualitative methods

## Abstract

Concerns regarding the welfare of farm animals continue to grow. Traditionally, research efforts have largely focused on refining existing management practices to improve welfare. However, the incorporation of views from those directly involved in animal care is equally, if not more, important. This study investigated the perspectives of Canadian dairy farmers on animal welfare. We conducted 16 interviews with a total of 22 participants from four provinces across Canada. Recorded audio files and field notes were transcribed, anonymised, and coded using deductive and inductive thematic analysis. The interview data revealed two major themes: (1) animal dimension of animal welfare, including views related to biological functioning, naturalness and affective states; and (2) dairy farmer identity, including, the voice of the ‘city’, what it means to be a good ‘cow-man’, and the nature of human-animal relationships. Dairy farmers emphasised biological functioning, but they made numerous references to the emotional and natural living aspects of their animals’ lives. Our work also provides evidence that farmers believed it was their duty to care for their animals beyond simply milking cows and making a profit. In terms of the larger debate, this study identified potential shared values with members of the public: opportunities for natural living and agency, attentiveness to individual animals, and the value of life over death. Finally, the emotional relationship that farmers developed with their animals highlights the values dairy farmers have for their animals beyond simply utilitarian function. Overall, these shared values could contribute to constructive dialogue.

## Introduction

Dairy farming in industrialised countries has undergone a process of consolidation for decades, resulting in fewer, larger and more productive herds (Barkema *et al.* 2015); a process that has increasingly failed to resonate with evolving societal values (Alonso *et al.* 2020). There is now a plethora of evidence suggesting that public citizens are increasingly questioning the care provided to dairy cattle. Although both citizens and those working in agriculture, including farmers and veterinarians, place great value on farm animals maintaining high standards of health (Cardoso *et al.* 2016; Balzani & Hanlon 2020), stakeholders that are removed from the farm increasingly want assurances that farm animals have a good life (Yeates 2017), including placing specific emphasis on aspects of care that promote naturalness and positive emotional states (e.g. pasture access and behavioural freedom (Vanhonacker *et al.* 2007; Jackson *et al.* 2020; see review by Placzek *et al.* 2021).

The growing disconnects between standard dairy industry practices and evolving societal values have resulted in some dairy organisations, such as in Canada and the US, adopting industry-led assurance programmes (e.g. Canada’s ProAction [DFC-NFACC 2018]; USA, FARM [NMPF 2020]). However, despite the widespread adoption of these programmes, many stakeholders, most notably the animal protection movement (Shields *et al.* 2017) and the public (Clark *et al.* 2016), may remain concerned about the care provided on dairy farms, particularly if the standards are viewed as being slack in order to ensure that the majority of farms are viewed as compliant (Weary & von Keyserlingk 2017). These concerns have also triggered discussion on whether the industry is able to maintain the public’s trust in the long run – an integral component of retaining social licence (Rollin 2011; Bolton & von Keyserlingk 2021).

An important and often under-utilised approach to finding solutions to challenges such as the disconnect described above, is to first identify the perspectives of different stakeholders before moving to discussing more contentious topics (Friedman & Himmelstein 2006; Rutledge 2009). For instance, in a recent interview-based study, Irish dairy farmers demonstrated a willingness to make changes to how they have traditionally cared for male dairy calves but at the same time were reluctant to consider rearing these calves for beef on their own farms (Maher *et al.* 2021). Surplus dairy calves (those not needed for milk production), were the centre of discussion in a recent focus group study with Canadian veterinarians. Although the veterinarians agreed that failure to find alternative solutions to the current methods of care place the dairy industry at risk, they highlighted a desire to help identify solutions they hoped would result in improved calf care, including: helping educate farmers about care and working collaboratively with multiple stakeholders (Hendricks *et al.* 2022). It is important to incorporate the voices of those involved in animal care and who have a shared interest in dairy farming, including farmers and veterinarians, as arguably this will help identify solutions that improve animal welfare (Dawson *et al.* 2016; Dolby & Litster 2019). Indeed, some have argued that the absence of farmers in the public debate on farm animal welfare is a fundamental problem (Meijboom & Stafleu 2016). Attempts have been made to understand how veterinarians and animal scientists view cattle welfare and to understand their roles, as well as others, in resolving problems (Ventura *et al.* 2016; Wynands *et al.* 2021). However, understanding the perspectives of farmers in North America has received less attention.

Previous work undertaken by our group describes the views of Canadian beef (Spooner *et al.* 2012) and pig farmers (Spooner *et al.* 2014a,b) on animal welfare. Interestingly, in the case of the beef farmers, the majority of participants clearly demonstrated an ethic of care, including a strong interest in doing the right thing for the animal (Spooner *et al.* 2012). In addition to striving for high standards of health, the beef farmers also insisted that a good life for beef cattle requires that the animal live in ways that promote natural behaviours (Spooner *et al.* 2012). In contrast, our interviews with Canadian pig farmers conveyed deep-rooted values that focused almost exclusively on the promotion of production and health, with little to no regard for natural behaviour or the emotional states of the animal (i.e. affective states) (see Spooner *et al.* 2012).

The Canadian dairy industry involves production practices that vary in the extent of naturalness, with organic farms incorporating pasture at least for some periods of the year (Smid *et al.* 2020) to tie-stall farms that provide little to no freedom of movement. In recent surveys, in Canada, about 73% of the 9,952 farms were tie-stall with the majority of the remaining farms employing free-stall housing and the average milking herd was 100 cows and 98% were family owned (Agriculture and Agri-Food Canada 2021). A dairy survey reported that approximately 30% of Canadian dairy farms provide lactating cows access to pasture, and approximately 60% of farms provide dry cows access to pasture (Smid *et al.* 2020). In relation to current housing practices addressed in the other two companion studies (beef; Spooner *et al.* 2012) (pigs, Spooner *et al.* 2014a), practices used in the Canadian dairy system are intermediate.

Thus, the aim of this study is to understand the views of Canadian dairy farmers on animal welfare, to provide an in-depth picture of their beliefs, values, and attitudes regarding suitable lives for dairy cattle under their care. The outcomes of this study will contribute to a growing body of literature on the public debate on animal agriculture and, specifically, dairy farming; our hope is that it will provide constructive input into current and future animal welfare policy discussions by including the voice of farmers. Improved understanding between on- and off-farm stakeholders will help to come to mutually agreed and satisfactory solutions.

The overall goal is to be able to understand how views vary among different segments of the Canadian population, and how the conflicts related to animal welfare of food animals may be resolved, as a basis for more satisfactory national, international and corporate policy. As this was a study that crossed multiple animal sectors and members of the public, we needed to ask questions about topics that would apply to all participants.

## Materials and methods

The Behavioral Research Ethics Board at The University of British Columbia approved the study (protocol #B06-0595). This study focused on Canadian dairy farmers with interviews conducted in 2009 and 2010; all 22 participants were directly associated with primary dairy production. Participants were recruited through a purposive sampling strategy designed to include those associated with different types of production and units of different sizes. Prospective participants were identified by several ‘key informants’ (Hammersley & Atkinson 2007) knowledgeable about the sector, directly by the researchers, and via dairy industry newsletters. Key informants included dairy veterinarians in various regions, dairy association leaders, and dairy science researchers. The researchers had no previous interactions with participants. Initial contact with prospective participants involved provision of a project summary and letter of initial contact which outlined the project and the interview process. If they agreed to participate, then they were given a consent form which was reviewed and signed at the interview. All documents outlined the purpose of the interviews: “develop an in-depth understanding of the values, beliefs, attitudes and ethical concerns of various groups of Canadians (livestock producers, rural and urban public, and members of animal advocacy organisations), about the appropriate care and treatment of agricultural animals” and potential goals: “promote a more inclusive discussion among major stakeholders. The work should also form a basis for reducing the conflicts that are beginning to emerge with the introduction of different programmes of animal welfare standards.”

### Participants

Interviews covered farmers from a range of locations across Canada and whom had a range of types of dairy systems and farm sizes. Sixteen interviews were conducted involving 22 participants. Interviews covered a total of 17 dairy farms because four interviews involved both the husband and wife, one involved father and son (who worked together), and one involved father and daughter (both owned their own farms). Of the 16 interviews undertaken, there were 16 males and six females. Participants produced milk in British Columbia (9), Alberta (4), Ontario (2) and Nova Scotia (1). Fourteen operations used free-stall systems, two used tie-stalls and one used a combination of indoor and outdoor ‘packs’ (loose-housing system, with a deep compostable bedding pack). Five operations allowed cattle access to pasture at some point in the year, one was certified organic, and another participant operated a dairy with both goats and cows. The number of animals associated with each operation at any one time ranged from 40 to 250 milking cows (mean of 122) and 80–350 animals in total (cows, heifers and calves). One participant also mentioned that they also owned a cow-calf ‘beef’ operation. All participants owned and operated their own independent farms (sole proprietorships, family-operated). Nineteen participants had grown up on dairy farms and were at least second-generation farmers, two had been involved in dairying for more than a decade, one was unknown.

### Interviews

Interviews, which lasted 1–2 h, were all conducted face-to-face (n = 15) with the exception of one which was done by telephone. Two interviewers (CS and JS) conducted all (except one) of the interviews, with one interviewer acting as the lead while the second limited their questions to supplemental probes and follow-up questions when further clarification was viewed as being helpful to the discussion. All interviews were recorded and transcribed (*verbatim*) by CS and hired professional transcribers. Professional transcribers were provided with an outline of goals of transcription, detailed information on style, and potential terminology spoken by participants. In addition to the recording, notes were taken during the interviews by both interviewers.

At the beginning of the interview, before the recordings were initiated, participants were given a brief summary of the study where they were told that we were interested in understanding their views on dairy farming, specifically animal care. They were then asked to review and sign a consent form, approved by The University of British Columbia Behavioural Research Ethics Board, stressing confidentiality and the right to withdraw from the study at their discretion at any time, although none withdrew. Participants were then explicitly told when the recording device was turned on and the interview was initiated.

During the interviews, participants were invited to respond to an open-ended, semi-structured schedule of questions that had been pilot-tested by three student volunteers involved in farm animal welfare and production (see Spooner *et al.* 2012) and questions were altered slightly over time as interviewers learned from previous interviews. This was done to ensure questions were clear and meaningful for participants. As a way of allowing participants to become comfortable in the interview setting, the initial set of questions covered demographic details, current farm animal operations, personal farming/production histories, and whether participants consumed animal products (all did). After these initial ‘ice-breaker’ questions, a series of questions were asked, specifically intended to elicit an in-depth understanding of the meaning of animal welfare (see Table 1). The main research questions were: what are the various views and ethical concerns of farmers regarding the welfare of dairy cattle and welfare in general; how are these views operationalised in dairy operations; and how do dairy farmers perceive their role in relation to other stakeholders such as members of the public? Collectively, the questions were designed to encourage participants to reflect on what ‘farm animal welfare’ meant to them, including any views on animal welfare beyond those specifically involving dairy production. In addition to the main questions interviewers followed up with pre-prepared probes, where needed, to gain a deeper understanding and to ensure their understanding was accurate.

**Table 1.** Open-ended questions that were used to facilitate discussion by Canadian dairy farmers on their views about animal welfare

Topic of interest	Examples of probing questions
The meaning of farm animal welfare	When you hear the term animal welfare, what does it mean to you? Are there any elements that you feel are more important than others? If you were invited to assess a farm animal operation from an animal welfare perspective, what would be the most important indicator to you that animals were subjected to good or bad welfare practices? How close do you feel you come to maximising the performance potential of each of your animals? Are there other competing goals involved that might compromise performance? What do you regard as the animal welfare challenges in your sector/industry and what welfare concerns have you heard raised by members of the general public and how have you replied? In ideal world, can we ask you to imagine how you would design your own welfare-friendly operation, from start to finish, from the animal’s perspective?
How participants came to their views	What is your background? (e.g. is there a history of family farming?) What were the important influences on your current perspectives about animal welfare/care? Have you implemented any changes in your farm animal care practices? What was your motivation to change?
Perspectives on animal capacities and needs	Do food animals have the capacity to be happy and if so, can you explain what the term ‘happy animal’ means to you? How closely are emotional needs of animals tied to their biological functioning (health)?
Perspectives on others	What role do you think the following groups could or should play in discussions around farm animal welfare? Who should be at the table? Consumers? Farmers? Animal advocates? Government? Industry groups? e.g Food industry Media? Veterinarians?
	Can you shed some light on the nature of the emotional relationship or bond that a farmer has with his animals (in relation to clarifying this for members of the public who might view farm animals as ‘pets’)? When members of the public speak about animal welfare, they say they understand the importance of good health, but they are worried that in farming practices today the emotional needs of animals are not being met. How would you respond to them?
Perspectives on consumer behaviour and the market	To what extent should consumers have a say about production practices? What are your perspectives on emerging product lines that provide assurances about animal welfare? What do you think is the best way to assure consumers that their concerns have been addressed?
Personal experiences of farmers	Do you enjoy farming? Why do they farm and what do you like about it? Do you feel fairly compensated for the work that you do (economically and socially)? What kinds of interactions/discussions (related to your farm practices) have you had with members of the public or consumers? If you were to win a lottery tomorrow, would you continue farming? If so, would you do anything differently and what?

At the end of the interview, immediately before turning off the recorder, all participants were invited to volunteer any additional comments relevant to animal welfare. All were encouraged to contact the researchers to add any supplementary comments afterwards, if desired, although no follow-ups were received. Participants were asked for permission for follow-up contacts, and all agreed. Participants were invited to receive copies of reports arising from the interview(s) and all accepted.

### Thematic analyses

After completion of interviews, extensive field notes were collected and discussed between JS and CS. Interview transcripts were analysed from a constructivist approach (Kiger & Varpio 2020) and deductive qualitative content analysis was primarily used to analyse the text data using a systematic classification process of coding and identifying themes or patterns (e.g. Hseih & Shannon 2005). For example, researchers were interested in understanding farmer views on animal welfare based on pre-existing knowledge of scholarly definitions of animal welfare and knowledge of the dairy industry. However, little knowledge existed about farmer perspectives so any text that could not be categorised with the initial coding scheme would be given a new code as in inductive conventional analysis (Kiger & Varpio 2020). All authors were animal welfare scientists. Therefore, attention was required when interviewing and analysing data in order to minimise bias regarding preconceived notions of what farmers should consider in their understanding of animal welfare.

At the completion of all interviews, transcriptions were reviewed multiple times in conjunction with preliminary memos and field notes were conducted using a ‘constant comparison’ method (e.g. Glaser & Strauss 1967); i.e. participant comments were divided into segments and classified or ‘coded’ before being compared and grouped with similar or related comments from other participants. This technique involved three stages: assigning codes to pieces of the data (stemming from interview questions and probes but also novel data) through coding, grouping the codes into categories, and developing themes that group together related codes. Transcript analysis began with reading transcripts line-by-line and assigning short descriptors to pieces of data relevant to the research questions. Next, similar descriptors were grouped into categories to create a list of codes and subcodes. Finally, the codes were clustered by similarity to identify themes in an organised codebook. In an iterative process, the codebook was revised to ensure relation to the data and relevance to the research questions. This process continued until patterns were identified within the data and were subsequently identified as themes. While we did not set out to continue interviewing until we reached saturation, we did find that the majority of new information was gained in less than 15 interviews. The first author coded all of the transcripts with the finalised codebook using NVivo (QSR International Pty Ltd, version 12; https://www.qsrinternational.com/nvivo-qualitative-data-analysis-software/home). For reporting of the results, unique numeric identifiers (e.g. 124) were assigned to quotes from participating farmers. Square brackets (i.e. […]) were used to indicate when a quote was shortened or when we inserted explanatory information to ensure the meaning of the quote was maintained. We emphasise the diversity of, and connection between, themes brought up by our participants as opposed to the quantity. Quotes were chosen to represent key ideas and have been modified in length for clarity.

## Results and Discussion

**Figure 1.**
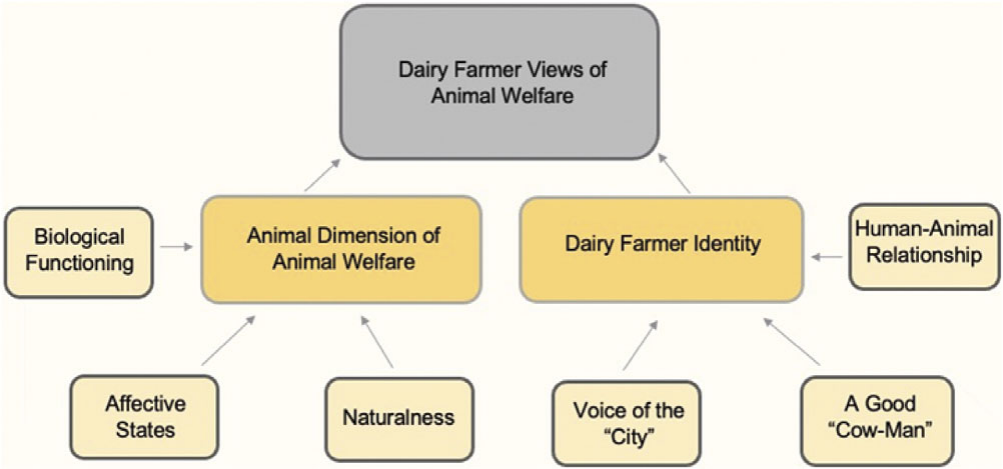
Thematic map of themes and sub-themes that arose from the 16 interviews (17 dairy farms) with 22 participants that set out to understand dairy farmer views of animal welfare (large grey box). The two dark yellow boxes represent two broad themes, and the six light yellow boxes represent sub-themes within each of the two themes. Arrows are used to demonstrate organisation of the topics.

This study is a qualitative inquiry using semi-structured interviews and based on a purposive sample of 22 dairy farmers, which set out to provide an in-depth picture of dairy farmers’ beliefs, values, and attitudes regarding animal welfare on dairy farms. Two broad themes were derived from the dairy farmers’ interview data and interpreted in light of our research questions, methodology and underpinning epistemological framework: the animal dimension of animal welfare and dairy farmer identity. Within each of these major themes, three sub-themes were identified. Please see Figure 1 for a map of the themes and sub-themes that were derived from the 16 interviews.

### The animal dimension of animal welfare

Sub-themes within this major theme fit with existing industry-wide concerns: importance of cow health, cow comfort, and reduced stress. They also reflected what we know about people’s perceptions of animal welfare as put forward by Fraser *et al.* (1997): health and biological functioning, affective states and natural behaviour. However, biological functioning was prioritised.

### Biological functioning

All of the dairy farmers that participated in the interviews placed great value on maintaining the health of cattle. Examples included providing proper nutrition, preventing disease and lameness, longevity, achieving high reproductive rates, and reducing stress. In addition, they emphasised environmental factors such as providing a reasonable stocking density, an appropriate thermal environment and a well-ventilated and clean facility. Te Velde *et al.* (2002) also found that many livestock farmers consider that attention to the animals’ biological needs achieves good welfare – including thermal regulation, dry bedding, disease monitoring, and adequate feed and water. One farmer, when asked to prioritise his management goals, stated that: *“I think priority for us right now is biological. You have to have a healthy animal first. Normal reproductive rates, long life expectancy that also pays off in the end, right? If you have an animal longer, keep it healthier, have more offspring, you benefit from it. I think it all interacts, and it all works together”* (214). In the words of one participant: *”Animals need to have access to food and water at all times… So, they have to have… a proper diet that works for their job"* (258). The weighting on biological functioning and health outcomes as the primary proxy for animal welfare used by farmers has been shown by others (i.e. Skarstad *et al.* 2007; Balzani & Hanlon 2020). Reasons given for this approach are likely explained, at least in part, because it is a pragmatic one: it strongly emphasises factors that are valued when seeking maximum production (and arguably economic return) (Te Velde *et al.* 2002; Lassen *et al.* 2006; Verbeke 2009; Kauppinen *et al.* 2010; Silva *et al.* 2013).

The concept of good health was often discussed in relation to reducing the incidence of disease or, if possible, preventing it all together. Specific diseases frequently mentioned by our participants included mastitis in cows and pneumonia in calves. For example, one farmer reduced mastitis by milking more times per day: *“…they’re milked four times a day – they got only twelve litres – that’s way more relaxed on her udder too and healthier also”* (194). The emphasis on health also extended to calves as relayed by participant 166 who stated that *“…if* [calves are] *always looked after, if they’re vaccinated when they need to be vaccinated, they’re not going to get sick. So, it all goes to better production."*

To protect against disease most of the participants spoke about immediately separating the calf from the dam and giving their calves colostrum in a timely manner. That farmers emphasised the importance of colostrum management in mitigating calf diseases on dairy farms has been previously reported (Sumner *et al.* 2018). However, other focus group work done in our research group reported that veterinarians are frequently sceptical as to whether colostrum management on dairy farms is carried out according to best practices (Sumner & von Keyserlingk 2018; Hendricks *et al.* 2022).

Immediate separation from the mother was considered necessary for calf health and to minimise disease transmission, as this practice facilitated individual feeding and monitoring of the calves, and environmental control. As one farmer stated, “*You got the calf suckling on mom, mom is in an environment where there’s bacteria, baby calf has got no immune system…. So, leaving the calf with mamma to suckle, getting their hair from the stomach – you’re asking for trouble”* (266). It is generally believed by the dairy industry that early separation provides benefits in terms of health, particularly for the calf (Neave *et al.* 2022). However, a recent systematic review of the available research on health benefits associated with prolonged cow-calf contact, has now called into question whether there are indeed health benefits to early separation (Beaver *et al.* 2019). Focus group and interview-based work on farmers and veterinarians has conveyed some resistance to reviewing this common practice of separating calves immediately at birth (Ventura *et al.* 2013; Neave *et al.* 2022). However, there is now considerable interest, primarily in Europe, on understanding the effects of prolonged cow-calf contact in dairy systems (Johnson *et al.* 2016; Mutua & Haskell 2022).

Many of our participants emphasised ventilation and temperature control to promote health, fertility and production. For example, *“We have fans, and a very open barn. Cows hate heat, and that’s our least profitable time of the year. Last year, when it was 40 degrees here, yeah, we lost a few cows”* (272). Recognition that an increase in the number of hot days can negatively impact dairy cattle welfare and production is now a generally accepted concern by dairy industries around the world (e.g. Gauly *et al*. 2013; Lacetera 2018); a fact that was also generally accepted by our participants. For instance, one farmer pointed out the challenge imposed by increased temperatures on meeting milk quota requirements which requires year-round calving: *“So when you have to ship so much milk per month… one of the hardest issues that we have is to try to keep cows bred so you’re having enough milk all the time because when cows are stressed under, especially… real hot conditions, we have trouble getting cows bred”* (234). One farmer also projected their feelings about what it would be like to be transported under extreme climatic conditions: *“We don’t take animals to the auction if there is a snowstorm, because they’re exposed to too much cold and bad weather. It’s not good for the animals… I would like not to be transported in extreme heat or extreme cold”* (258).

Typically, comfort was associated with physical comfort and often in relation to stall design. Goals for stalls included keeping them clean and dry with deep soft bedding or ‘comfort mattresses’ so cows felt comfortable to lie down and large and wide enough for cows to easily get up and down. For example, “*We put a lot of efforts in cow comfort. Clean stalls and lots of bedding*” (194).

Many of the participants emphasised lameness as one of the biggest problems in dairy production. For example, one participant stated that, *”I don’t know many farmers that aren’t concerned about the lameness thing. So, it’s already a concern and we have research galore trying to solve some of these problems”* (222). Another participant recounted that, “*Lameness is an ongoing concern in our industry. We try to minimise lameness through our feeding programmes and our regular health programmes”* (228). In contrast, this farmer suggested some causes being out of their control: “*We try to provide an environment for them where we try to minimise laminitis, but there are certain metabolic or anatomical issues that we have no control over”* (228). That lameness was recognised as one of the greatest challenges facing the dairy industry at the time when these interviews were conducted can be seen as both positive but also worrisome. That farmers struggle to identify lame cows on farms has long been recognised. For example, two studies carried out over a decade apart, Espejo and Endres (2007) and Jensen *et al*. (2022), both reported that farmers were only able to identify, on average, one in three lame cows. Despite challenges associated with identifying lame cows, the fact that our participants recognised that this malady is a serious problem has enabled research on identifying what types of changes or interventions minimise lameness on farms. Work has reported that benchmarking may be one important tool to increase farm engagement to improve animal welfare on farms (lameness: Chapinal *et al*. 2014; calf feeding: Atkinson *et al.* 2017). What is worrisome, however, is that despite great efforts by researchers and dairy industry organisation assurance programmes that focus on lameness, there has been little to no improvement in lameness prevalence over the last decade; globally, lameness prevalence is estimated at 23% (Cook 2017). Lameness has been defined as a blurry, ill-defined concept among both farmers and dairy consultants (Olmos *et al*. 2018); a fact that may make it a particularly difficult challenge to overcome.

Reasons provided by participants that may provide some insight into why lameness continues to be a challenge arose as a result of conversations amongst our participants concerning types of housing, with some blaming ‘other’ housing systems that differed from their own as being the reason why this problem has remained unsolved. In the words of one tie-stall barn farmer: *“Now this is one big fault with these free-stall barns. Because under our cows we keep lots of bedding and they’re dry. The free-stall barns, no matter what you do, you walk in the back it’s going to be wet. There’s no way to eliminate that… your cow’s foot is always going to have moisture on it and wet. And that causes lameness too”* (234). The presence of cognitive dissonance (see Festinger 1962) has been used to explain, at least in part, that these types of arguments in essence place the blame on others; as in this case the tie-stall farmer conveying that lameness is a ‘free-stall’ housing problem. Similarly, others have reported that dairy farmers using pasture-based farming systems shared a belief that lameness was a concern limited to intensive housing systems, and thus not a problem on their farms (Olmos *et al.* 2018). Interestingly, Bran *et al.* (2018) reported that the lameness prevalence on 44 pasture-based dairies in the same region was 31% and was indeed a serious welfare concern.

One factor that may contribute to continued challenges in the industry with regard to prevalence of lameness may relate to the view that farmers see their farm as a whole system: where a small percent of lameness should be regarded as a success in a system that cannot be perfect. This may conflict with how members of the public view farms: who attach greater significance to poor welfare of individuals (Spooner *et al.* 2014b). These different frames of producers and citizens may parallel those of conservation biologists (focusing on animal collectives) and animal welfare advocates (attending to individuals) (Paquet & Darimont 2010).

In addition, beliefs around giving animals a chance to recover, rather than culling, may also come into play. Decisions about culling cows were complex and highlighted areas of disagreement amongst farmers with regards to their views on the role of longevity in cow welfare and perhaps reflective of fundamental difference in views on life and death. Questions were raised about whether farmers should nurse cows back to health or carry out prompt culling. For example, one producer felt that reduced longevity of cows in the current industry was reflective of poor welfare: “*This is the most incredible, staggering thing… in our cows, the average lactation might be four lactations… Whereas they could live to be — we have cows that are, twelve, at least* (258). This may also overlap with concerns of the public who may also value life rather than death. In contrast, another producer felt the opposite: “*They* [the public] *want to keep the cows around a little longer. Is that good or bad? The people always say, ‘Oh how old are your cows?’ They think if I have really old cows, then I’m a well-managed farm. The old cows tend to suffer more. It’s like old people. Keep the young around — like teenagers, they’re always healthy!”*

Culling decisions were also emotionally difficult for farmers but they recognised that they also needed to detach. For example, one participant described a neighbour who could not kill his own animals: “*We have a neighbour guy who calls somebody to do it for him because he just can’t even. Well, I go there and shoot them"* (246). Another producer said: *“Cows, they can go ten, twelve years, around here, but, you know, you got your favourite cows, but bottom line is, you can’t keep everyone, right? It’s hard, but you have to accept the fact that they’re all going to go someday”* (256). This last quote also illustrates the necessity of ‘detaching’ or preventing the development of strong emotional attachments. In part, this necessity stemmed from the fact that producers were running a business that involved culling cows and because emotional attachments were distressing.

### Affective states

Participants also made references to concerns surrounding the emotional states of the animals under their care. They also described animals as displaying emotions. For example, one farmer conveyed their thoughts in terms of a self-directed question, *“A good life for one of our animals? … I mean, an animal that is not gonna encounter suffering… to be healthy, not overcrowded, not in pain, not scared, not to have to be in fear of anything”* (274). That farmers hold a sensitivity towards pain in the animals under their care has been reported previously (Wikman *et al.* 2013). In the UK, dairy farmers agreed overwhelmingly that lameness is associated with pain and suffering (Leach *et al.* 2010). This quote also illustrates negative welfare language and an emphasis on achieving good welfare through minimising negative emotional states. This narrative reflects a ‘political narrative’, dating back to the 1960s, where farmers focus on eradicating negative welfare rather than incorporating and promoting positive states, more common in recent discourses (Muhammad *et al.* 2022).

All participants spoke about the importance of minimising stress in their animals. The term ‘stress’ was used in a variety of contexts and particularly in relation to human-animal interactions. Further research would benefit from probing more deeply into the various meanings of stress in relation to animal welfare. The available information on dairy farmer attitudes to animal stress is conflicting, as some studies report that farmers acknowledged that mitigating stress is important (Vanhonacker *et al.* 2008) but others report that it was not brought up by farmers (Te Velde *et al.* 2002; Bock *et al.* 2007; Kauppinen *et al*. 2010). In beef production, low stress handling was strongly emphasised by farmers (Spooner *et al.* 2012). In the current study, several farmers mentioned low stress handling when describing how they loaded cattle onto trailers for the journey to the slaughterhouse. One farmer described this from the perspective of he himself being a cow*: “When it comes to slaughter… As much as possible, we like that to be a calm, stress-free transition from the barn… to the truck. …I would say if I was the animal, I would appreciate not having to, you know, be beaten and chased and loaded into a truck, under stressful circumstances…”* (258).

For many farmers the goal of healthy animals was closely linked with reducing stress and, in turn, increasing production. References to low stress handling techniques included phrases about being calm, quiet, and not rushing or hitting the animals: *”You don’t make quick movements around them, you’re slow, you do what you can not to startle them and things like that"* (222), or *“…you conduct yourself in a way that is not stressful to the animal. We don’t appreciate people screaming, hitting and swearing at animals… that stresses an animal, and if an animal’s stressed, it doesn’t function as well”* (258). The low stress handling approach was also linked to improved production. In the words of one farmer, *“If the animal’s not happy, if you’re rough with the animals, whatever it is, they’re not going to produce”* (166). Similarly, *“Stockperson handling is very important. You can get some herds … – they’re handled roughly – … but I always figure that costs money, because… when she’s stressed, she’s not letting her milk down”* (170). Increased fearfulness in dairy cattle has been previously reported to be negatively correlated with milk yield and quality (Breuer *et al.* 2000; Hemsworth *et al*. 2000). A number of farmers also described good animal handling as being associated with good health. For instance, one farmer who made use of anaesthetics for dehorning his calves justified this practice as they believed that this was an important step to maintaining health: “*Use a pain reducer just to make sure they continue eating type of thing, so they can get healthy”* (194).

Several farmers also linked low stress handling with respectful treatment of the animals: *“I want them to die respected and it bothers me to see – it was a movie on television, how they died with the – up the ramps, stun gun, fear, you know, that bothers me. I would rather shoot them in my own yard where you know they’re calm and quiet and they’ve lived and they’ve died well”* (274). This quote also illustrates an empathetic response and moral responsibility of the farmer to their cows. Interviews with pig farmers have previously reported that a farmer’s ability to empathise with their animals is a key factor contributing to how they handle their animals (Coleman *et al.* 2003), but Phillips *et al.* (2009) argued that factors such as facility design also play a role.

Although we did not specifically ask about disbudding or dehorning during the interview, these topics were raised by eight farmers. The reason that this topic was salient amongst many of the participants may be due to the timing of the publication of the first version of the Canadian Code of Practice for the Care and Handling of Dairy Cattle in 2009 (National Farm Animal Care Council 2009). This industry-led ‘Code’ specifically stated that pain mitigation was a requirement when removing horns from dairy cattle on Canadian dairy farms. These participants acknowledged that dehorning was necessary for the safety of the calves and handlers and six (of eight or 75%) used a combination of sedative and local anaesthetic, one was planning to do the same and another used a non-steroidal analgesic: “*You can dehorn them for their safety and health as well as for the operator… but at the point of doing dehorning, you have a responsibility to minimise pain in those situations when you’re handling them"* (222). A recent national survey of dairy farm calf care practices in Canada (n = 891) reported that 86% of farms disbudded their calves using cautery and, of these, over 60% reported using local anaesthesia (similar to our participants) and about a third also stated that they used non-steroidal analgesia (Winder *et al.* 2018). Given public concern for painful procedures (Spooner *et al.* 2014a; Robbins *et al.* 2015) and the existence of a solution, this is likely to remain a conflict between farmers and the public.

Although half the farmers used the term ‘happy’ as a descriptor when talking about their animals, this term was frequently linked to conversations around whether the cow had good welfare and was producing well. For example, “*For us, happy animals produce*” (166), or “*I see a cow- she’s chewing, we’re milking her and she’s chewing her cud, her eyes are like” ahh," she’s in wonderland, she’s daydreaming or whatever she’s dreaming about, that’s a happy cow"* (272). Some participants also made inferences about the emotional lives of their cows, using words such as ‘frisky’ or ‘playful’, and ‘calm’, but also in relation to specific behaviours such as scratching on a brush or grazing on pasture: “*If I open up the gates after milking and the cows run… they play around and they lift all the four legs from the straw, I think then you have happy cows”* (304).

However, some participants were uncertain about using this term, citing concerns that it was being anthropomorphic: *“My cows are happy sure they are. But it’s just not terminology that we use here in the industry. So, happiness is more like a human being thing”* (228). Critics of the animal rights movements have previously been noted to frequently denounce anthropomorphism, arguing that it is inherently wrong to ascribe human sentiments to animals (Bruni *et al.* 2018). Along this same line some participants questioned whether cattle were capable of experiencing a wide range of emotions. For instance, one participant stated that: “[Cattle] *understand fear, they understand pain, how they express that and whether we as herds’ people or farmers can understand and recognise how they display that is the next question… Whether they can express joy or happiness is something I can’t tell you*” (246). In some cases, participants appeared to be more comfortable attributing other states such as being ‘comfortable’ or ‘content.’

There were many examples where farmers described cows in anthropomorphic terms *(“day-dreaming”, “one little cow just hates me”),* which might offer shared values with members of the public. Perhaps farmers are not so different. For example, many farmers spoke about introducing mechanical brushes and how ‘happy’ cows were when using them to scratch. Although one farmer acknowledged that the brush no doubt brought pleasure to the cow, it did not translate into profit: “*Adding a brush in the barn where they can scrub each other or themselves… It’s something… that you can’t translate into more money, but it’s more comfort for the cow, she’s better at ease, its less stress, there’s no itching, so she feels better"* (194). Similar to pasture (see below), access to brushes was good for welfare but not necessarily for productivity and profit.

### Natural living

Little attention was given to the natural living construct of animal welfare (Fraser *et al.* 1997), with the exception of whether cows should have access to pasture and, to a lesser extent, social contact for calves. In both cases, however, many participants were reluctant to embrace these methods of care due to their perception that either production (in the case of pasture) or health (in the case of calves) would be compromised.

In general, farmers felt there were some benefits to allowing cattle access to pastures (freedom, grazing, sun, good for hooves, public perception) but there was still ambivalence. Keeping cows on pasture had limitations that were overcome by ensuring cows remained indoors with controlled environments, nutrition and social interactions; thus, indoor systems were often viewed as a suitable or a better substitute for outdoors. This view lay somewhat in-between the views of pig and beef farmers. In comparison to our companion papers, dairy farmers echoed some of the views of pig farmers where they expressed industrial values with an emphasis on productivity achieved by science and by overcoming the limitations imposed by nature (Spooner *et al.* 2014b). In contrast, dairy farmers differed from beef ranchers who strongly believed providing a natural grazing system was better for the cattle (Spooner *et al.* 2012); likely a reflection of beef rancher agrarian values that view animals as part of a natural, land-based system that was worth preserving (Spooner *et al*. 2012). In the case of dairy farmers, the value of pasture had more to do with dairy welfare than grazing as part of preserving ecosystems.

Of all of the participants (eleven of 22) who mentioned pasture, all but one agreed that there were positive aspects to having cattle out on pasture. However, there were clear differences in beliefs amongst our participants as to whether providing pasture was needed for a cow to have a good life. A few farmers stated that they allowed at least some of their animals access to pasture at some point during the year and they conveyed their beliefs that pasture provided health benefits. For example,

*“A lot of people’s cows…* [are] *just in the barn, they go from this pen to that pen and that’s their life. From the day they’re born they’re in the barn or pen and they’re never really outside, they’re in these free-stall barns, and don’t get me wrong there’s nothing wrong with these free-stall barns. But I think our cows are happier because they go out, they graze like cows did years and years ago, they come in the barn, they get fed silage in the barn, so if they want to eat in the pasture, they go out across the road here and there’s the woods over there… They don’t mind being rained on and that was a kind of a revelation to me”* (234).

Whether or not some farmers used pasture depended, in part, on the weight they gave to what they perceived as benefits and how much they valued productivity over welfare. For instance, one farmer stated that: *“Pasture in the summer when the sun’s shining…Yeah…* [cows] *do better. They don’t always produce more milk, but is that the end goal*?” (246). This quote also illustrates an example of when productivity is not dependent on good welfare. Many participants also pointed out several limitations or barriers that prevented access to pasture, including decreased milk production, economic losses, geography, climate, and lack of available land base. For example, *“A happy cow you would have basically free range, a nice grassy field… But, in reality, that’s not always the way it is anymore, to get top production. And you know what? It still comes down to – this is a business. You have to do what you think is best to run your business efficiently and to get maximum productivity”* (166). Similarly, as also reported recently by Smid *et al.* (2022), some farmers spoke of the economic challenges of putting cows out on pasture: *“Pasture is awesome if you can do it, but it’s just a matter of land and cost and what people want to pay for milk. There are some guys around that do it… but mainly smaller – like, our size of a farm, we just don’t have the land base”* (170). One farmer who had no means to provide pasture access felt that they had a responsibility to make sure indoor housing was comfortable: *“Access to pasture – there is none – because of economics. But then with that also increases your awareness and responsibility towards cow comfort etc”* (228). Differences in motivating factors amongst farmers were reported by Hansson and Lagerkvist (2016), where they found that some Swedish dairy farmers included in their decision-making processes whether their cows were happy; not just whether the decision increased production and hence profits.

Providing access to pasture was also often raised simultaneously with challenges associated with restriction of movement. One tie-stall farmer who let his cows outside did so in the belief that this ensured their well-being and contributed to improved health: *“Well I think cows that are confined 24/7/365, you know, tied up is purely a convenience and crosses the line into animal well-being issues… It’s a tough one… I believe that if they have… reasonable access, it doesn’t have to be 365 days of the year, but to an outside environment… then that’s a positive for you for sure”* (222). This sentiment of pasture being good for animal health was also echoed by another participant: “*Bring them all in, leave them inside and feed them inside – that makes more sense, but the health of the animal, I think they need sunshine just as much as we do”* (246). More recent work in Canada reported that Western dairy farmers were divided on whether pasture access was good or bad for health. Those that incorporated pasture firmly believed that it was beneficial for cow health and those that did not cited beliefs that indoor housing allowed them to maintain good udder health (Smid *et al.* 2022).

Several farmers believed that the cow ‘knows best’ and therefore should be provided environments where they can choose to alternate between coming inside and going outside: *“If you have free choice they’ll go out after four or five at night, but they don’t want to go out at all during the day because it’s scorching hot and they just suffer actually in the heat badly, so we have them inside in the day and have the fans on in the summer-time and, you know, a breeze continually going through that barn at a cost to myself and then outside at night when it’s cooler and no sun torture”* (222). In contrast, other farmers justified their zero grazing approach based on their perception of what they felt the cows would want. One farmer conveyed that despite trying to use pasture his cows did not want it: *“Access to pasture – in a perfect world I would love to shoo those cows out there every single day and let them run around for an hour – but 90% of the cows would be home in half an hour wanting the* [Total Mixed Ration] *and their comfortable beds and the shade”* (272). Others argued that given indoor housing systems met, albeit not perfectly, the cows’ need for safety, food, shelter, water and social contact there was no need to provide pasture: “*Well, what do humans want and need? You need shelter, you need food, you need buddies… I’m providing that… I’m not an expert on the emotional state of the cow, but even out when they were grazing, you know they had the food source, they had the water, and you had your herd-mates around you, and I think we kind of mimic that to the best of our abilities here, it’s not perfect… you know there’s no predators, you have adequate food, there’s adequate water, you never undergo drought…”* (266). Farmers frequently included concerns about potential losses in production when cows were given access to pasture: “*I can’t chase my cows in the pasture because I will lose a lot of milk and I will lose a lot of growth from the field, because Alberta is too dry. What we do is, all the young stock are raised on pasture… But it’s something we want to* [do] *because it’s healthy for the cows. They grow better, they exercise, they are not overfed, they are always in nice condition… I see that a lot of farmers, a lot of people see our heifers walking here in front and I’m pretty sure they like it. I think it’s important, but I can’t speak for other guys”* (304).

Providing a choice of what types of environments the cows prefer, and when, including access to pasture, was viewed to be important by many of the participants. In the words of one: *“If the gate’s left open, which it is on our farm, because they make that choice themselves”* (246). Central to this discussion is the growing evidence that dairy cattle are highly motivated to access pasture but do so primarily in the evening hours (von Keyserlingk *et al.* 2017) and prefer indoors when the temperature humidity index is high or inclement weather (for a review, see Charlton & Rutter 2017). The ability to provide animals agency, to have more control over the way they act and to be able to learn about the world around them, and to make informed choices, is a rapidly developing field given its importance in promoting positive emotional states (Franks & Higgins 2012; Špinka 2019). Allowing animals some agency is an opportunity where farmers and members of the public may share values (Spooner *et al.* 2014a,b). Finding ways to increase agency in dairy farming may be important. For example, providing brushes, using robotic milking systems, allowing some form of cow-calf access, using positive reinforcement training for procedures (Lomb *et al.* 2021), free-stall systems, and choice for indoor and outdoor access.

Overall, there appeared to be tension reflected in the comments on pasture access, a view also raised by Smid *et al.* (2022) who held focus groups with Western Canadian dairy farmers on the topic of outdoor access for dairy cattle. On the one hand the majority of dairy farmers viewed pasture access as favourable for cow welfare. However, on the other, they also viewed that providing a safe, comfortable and healthy indoor environment was a good substitute or that it was not economically feasible. This may be an area demonstrating cognitive dissonance in farmers, where their authentic views of pasture are quickly trumped by rationales for why they cannot provide pasture. However, views about pasture also illustrate some potential shared values with members of the public: both having positive views about pasture and autonomy.

Although views differed about the importance of social contact with other milk-fed calves, the majority of participants individually housed their young calves due to perceived health benefits associated with reduced risk of disease transmission. In the words of one participant: *“We have our calves in hutches. And that kind of separates them. So… there’s a little bit of a conflict there, because I do agree that it’s nice to have them in a natural situation, but we’re also looking for the best health of the animal, too. And when you have the social contact, then you also have this threat of disease as well”* (214). Despite their beliefs, there is currently little evidence of a consistent relationship between individual housing and calf health, for reasons that likely have to do with the myriad of factors that impact disease risk thus making systems difficult to compare (for a review, see Costa *et al*. 2016).

The degree to which separation between calves was needed did vary with some stating that no contact was needed while others felt that some physical social contact was important to enable calves to prepare for their future social life: *“My calf hutches are not the recommended spaces apart. My calves can touch each other… there is never one by itself. In fact, if I have one calf born in one month alone, I’ll buy one so it has a friend… they learn a lot; you know from competing with each other for feed and stuff like that… if it’s kept by itself and then you put it in with a group where it’s got to mix sooner or later it’s not going to do well because it’s never had that previous exposure. And, you know, one calf tied by itself in a hutch, normally, it doesn’t like that. It wants a friend”* (222). There is now evidence that the challenges associated with social isolation during the milk-feeding period, including deficient social skills, fear of novel situations, (for a review, see Costa *et al*. 2016) are not mitigated by allowing limited physical touch (i.e. nose touching between bars) (Duve & Jensen 2012). Given new evidence, an important area of future research will be to investigate the different types of communication needed when seeking adoption of best practices that result in improved welfare on farms.

### Dairy farmer identity

Farmers often made comments about what it means to be a dairy farmer: their responsibilities and commitments, making a living, pride in their work, and their relationship with non-farmers (members of the public). Our farmer participants implicitly acknowledged that humans play a significant role in impacting the lives of the animals under their care, in large part because they believed that making a living as a dairy farmer is dependent upon good welfare. Our findings suggest that our participants held an overall strong sense of duty to care for the animals, an acknowledgment that they themselves play a key role in providing care and pride in dispensing that care. However, there were also numerous conversations noting that they believed that members of the public challenged their role as care-givers and that that they will likely continue to play a role in challenging their ethic of care.

### The voice of the ‘city’

All farmers, except one, had an open-door policy for their barn and many were involved in organised tours of various kinds and had many stories relating to interactions with the members of the public (described by one farmer [258], as the people from the ‘city’), who often challenged what farmers did. While there was acceptance by certain farmers that some of their practices may be viewed with concern by the public, in general, participants suggested members of the public were misinformed; a fact that led to some farmers becoming frustrated as they believed that members of the public expected perfection. They all felt that after they had shown people the barn etc, that people left feeling content that the animals were well looked after, and that they would be happy to drink their milk. In addition, they often made statements about wishing members of the public would be more self-reflective about their own lives in order to judge farming more fairly. Some participants fell back on anthropomorphism, expressing arguments such as, *“the pen where there’s sick and sore cows, and with 500 cows in our operation, there might be 3 or 4 max that are sick or sore that day. So I go, you show me a broad spectrum of society with 500 people where there aren’t 3 or 4 with a few ailments, right? But they put their own values onto my farm, and yet they sort of ignore the values that they have for their own animals, and their own children a lot of times, or their own parents”* (272). This quote also highlights differing levels of significance attached to specific individual cases of inadequate care, as identified in potential reasons for why lameness remains a challenge to the dairy industry.

For members of the public ‘3 or 4’ animals may in fact infer that many (potentially even millions when considering all farms) of animals are likely subjected to similar experiences in contrast to farmers insisting on the relative rarity of such examples (for additional discussion, see Spooner *et al.* 2014b). This insight could help respective stakeholders to better understand how others frame the issues.

Arguments concerning the public’s view of farming were also brought up when discussing lame cows and how best to treat them. Some farmers commented on temporarily putting lame cows onto pasture as a way of helping them but had stopped doing so after members of the public, driving by, had stopped to offer complaint about something being wrong with their cows. This ‘forcing of their hand’ by the public frustrated some farmers. For example, “*So she had some hoof issues that were going to take months to resolve. So, this cow was in pasture beside the road and the SPCA was contacted about this cow. Well, it was probably the best situation for her to be in at the time… she was, you know, kind of put out to pasture to heal, right? And so* [now] *if something like that comes up, it’s probably best to put that cow inside a barn on a bedding pack somewhere"* (246). However, this same farmer was sympathetic to the concern: “*you have to be able to explain that stuff. Yeah, you can understand people’s concern."* Many working in agriculture, including veterinarians (Sumner & von Keyserlingk 2018) and farmers (Ritter *et al.* 2020), argue that educating the public will lead to greater acceptance of current practices. However, as Ventura *et al.* (2016) showed, educating the public to dairy farming can also result in reduced confidence in the industry since this practice also increases awareness of all practices, such as cow-calf separation which is not viewed as an acceptable practice by the public (Sirovica *et al.* 2022).

Many participants recounted stories about conversations they had had with members of the public about why their cows do not go outside. As stated by one farmer: *“*[The public] *tell you that, “Why do you have the cows always inside and why don’t we have them in the pasture?” … then we explain…that it’s better to have the cows inside for – when they’re producing in the summer, it’s also thirty degrees plus Celsius and that’s too warm for a cow”* (194).

Some farmers raised concerns regarding what the public would say about calves being kept individually but argued that the public simply failed to understand why this form of calf housing was important. For example, “*They* [the public] *do not like the calf hutches, and they figure, those poor little things are in their huts, and then you explain to them how they get their own individual environment with the air and they actually do better in there, they’re only in there for a couple of months… And they don’t seem to understand that an isolated calf is actually a lot better. Like you can’t have a cow with its calf too long because of the disease transmission and the fact that it’s gonna get trampled or whatever. I mean, it doesn’t work”* (274). This quote also highlights the emphasis on disease transmission over other potential benefits of social housing.

There were many statements made that were rooted in the belief that animal welfare and production are intrinsically linked. These types of arguments often arose when participants were speaking against members of the public that were critical of farming; as animals that were not taken care of was bad for business. In the words of one participant, “*To me, it’s not mutually exclusive looking after the welfare of your animals and generating revenue and even lots of revenue off of them… They have to understand, from a farm point of view, that the economics of being indifferent or not doing those things, that it actually costs them a lot more”* (222), or “*So, that’s something that people don’t have any idea how to raise animals, but they come to the farmer, assuming the farmer is going to hurt the animals. They assume – there seems to be an assumption that farmers are mistreating animals all the time in order to make a living off them. Well, it just goes against intelligence, because if you do that, you’re out of business fairly soon, for one thing”* (258).

It is perhaps not surprising that farmers resented being criticised or told what to do by ‘city’ folks. Part of the reason the participants liked working as a farmer was that they were able to act independently. When asked what they liked most about farming, most farmers said they enjoyed the lifestyle: being their own boss, being near family, flexibility to spend time with family, working with animals, working in the field, having no commute, and breeding cows. For example: “*Being your own boss, filling your own time schedule. Working with animals, whatever changes you want to implement, you’re basically free to do… Well, then also the lifestyle, I guess, being able to have every meal with your family and sharing their time on the farm and — it’s something I really enjoy”* (192). There was often a sense of pride conveyed when they spoke about raising animals. For example, “*We’re not raising an animal for slaughter, we’re raising an animal that we’re gonna take the best care of that we can get the most amount of milk out of them – when its lifespan is done, then it goes but… So, I really enjoy that part of it”* (274).

As one farmer indicated, the pleasure of being your own boss has been diminished over time due to the growing public concerns about animal welfare. This change over time was viewed as troublesome given that people are now trying to tell them what they can and cannot do: *“Farming used to be a thing where nobody really told you what to do, but that’s part of the problem, animal welfare. Like, we don’t mind having a discussion about it, as long as it’s a discussion, not being told what to do. So, that’s the main problem of any type of, you know, dialogue between the city and the country”* (258). Farmers have previously described autonomy as a value that is accompanied by a specific lifestyle linked to farming (Niska *et al.* 2012) where one is one’s own boss (van Gelderen & Jansen 2006). However, the extent to which this value is fulfilled in farmers is constrained by the extent to which they are able to achieve remuneration (Stock & Forney 2014). In terms of conflict between the city folk and farmers, this emphasis on autonomy may also reduce farmer openness to self-reflection or analysis related to current animal welfare practices but it does highlight the importance of dialogue.

Interestingly, despite all participants selling their milk for the same price – as per the supply management system in Canada (Heminthavong 2018) – the views amongst our participants often differed with respect to whether they felt they made sufficient money. While some felt they were not sufficiently compensated: *“You’re basically not making money, it’s costing you to live, but you really love doing what you’re doing, so you just keep on going till the money’s gone”* (258), others felt that they were justly remunerated *“… one thing about dairy farming is it’s a good way to pay the bills, but there’s a lot of work involved with it. It’s not an easy – it’s a seven day a week, twice a day, sometimes three times a day, year-round”* (170). In all cases, regardless of remuneration, farmers enjoyed their work.

Most farmers mentioned that their jobs were difficult: they had long hours and big responsibilities, but they still chose to farm because they liked the lifestyle: *“I mean, sure it’s a great place to raise the kids and things like that. But you know what, I’ve got my cell phone with me twenty-four hours a day. The night-time milkers, when they’re milking, any problems, they phone me. Whereas some other people, when they’re done work Friday afternoon, they’re done till Monday morning. They don’t have to think about it again. For me it’s a lifestyle and it’s a good lifestyle and I enjoy it”* (166). When asked if they would continue farming if they won a lottery, all farmers answered ‘yes.’

### A good ‘cow-man’

Salient throughout the conversations was the notion that certain skills (e.g. observation, quick response) or qualities (e.g. attentiveness, vigilance, dedication) were associated with being a good farmer who provided good care to their cattle – in essence what it means to be a good ‘cow-man’ (266). Previous work has reported that a farmer’s view of person, role, and social identity are indeed dynamic and complex, but also context-specific (McGuire *et al.* 2013). Taking care of animals’ health and welfare is a corner stone requirement for having a ‘good farming identity’ (Butler & Holloway 2016).

Many farmers spoke about the importance of being attentive to individual animals and being vigilant to identifying potential problems, such as sick or stressed animals. In addition, they emphasised the importance of dealing rapidly with problems such as “*…Don’t leave a sick animal. You look after everything immediately and a lot of things before it ever becomes an issue. So, it’s recognising problems before they… become clinical”* (222). This same participant added that, “*Around here… we go sit half a day with a little calf to make sure it’s fed… We don’t lose calves. That’s kind of the way it goes, but if that means sitting with it and bottle-feeding it, petting it… you don’t leave the barn if one needs any medical care or attention”* (222). When cows are ready to calve, several farmers mentioned regular monitoring, but also implied that they believed standards differed across farms. For instance, one participant stated that: “*When a cow has to calf, even an easy cow, if it’s in the middle of the night, we always go out. So – and not too many people do that”* (304).

The road to becoming a good ‘cow-man’ was generally accepted as including learning through experience (e.g. growing up on a farm) or via training and/or education. The factors that contribute to what it means to be a good farmer has been the subject of much discussion (i.e. Sutherland & Darnhofer 2012). For some, this was tied to growing up on a farm as this provided long-term ‘lived’ first-hand experience: *“Being with the cows, it just becomes almost like second nature to you when you’re brought up like that. You can see an animal that is stressed or anything, you don’t have to learn that, like we really notice… with employees that have never been around animals. It takes years to be able to understand”* (274). Whereas for other farmers being a good ‘cow person’ was viewed as one where the individual had a natural ‘gift.’ Good observation skills were considered important but difficult to quantify. For instance, to be a good cow man, one participant stated that it required of them: *“To know an animal is sick, to know a cow or calf is sick before the cow or calf knows they’re sick”* (234), *or “… that cow there she just had a nice heifer calf yesterday, and I’d take a look at her and I’d say, jeez I think you’d better give her a little bit of calcium, she doesn’t look that good to me, I think she’ll come down with fever. ‘Why do you say that?’ and I’d say, ‘well just take a look at her eyes, she doesn’t look good in the eyes, or her ears are a little cold, or her skin just doesn’t feel right, there’s all kinds of things’”* (234). In this case specific skills were difficult to describe but required an innate ability to ‘read the cow’ as described by one participant: *“… a cow can’t tell you what’s wrong with her. You have to determine what’s wrong with her. It’s not like a human, ‘I’m sore here or here,’ you have to be able to read a cow”* (256). This account was similar to what Burton *et al.* (2012), referred to as ‘watching know-how’ coined by Dockes (Dockes & Kling-Eveillard 2006) as an essential quality of a good livestock farmer. This requires knowledge of specific animals in the context of their normal behaviour as well as general experience of how to work with cattle.

One farmer argued that new technologies place farmers at increased risk of being ‘distanced’ from the animal but even under these circumstances a good ‘cow man’ was key to being successful: *“…again, an animal isn’t a machine, it’s a living being… a lot is being diminished because of this automation…. And I still think on your most profitable farms… you’ve got a good cow man looking after things”* (266). Despite some reports of tremendous advantages of integrating technology on farms (Schillings *et al.* 2021), ethical questions have also been raised, including the notion of care and farmers’ identity as animal care-givers (Bos *et al.* 2018; Werkheiser 2018).

In terms of human-animal interactions during handling, one farmer spoke about the positive role of women as farmers: *“I love the girls, when they milk, they’re so calm, the guys – we make sure they’re taught how to handle cows, how it’s safe to handle cows, like we don’t want them doing anything stupid. Don’t chase them, don’t hurry, just pretend it’s your mother and your mother’s doing it, okay? Don’t stress them”* (272). That farmers perceived women as having special competence when it came to caring for animals was also reported by Heggem (2014) who interviewed 17 male and ten female Norwegian farm owners and noted that women were more likely to state that they were drawn to farming because of the animals and men acknowledged that they were better milkers.

### The human-animal relationship

All of our participants commented on their relationship with their animals: some speaking about their emotional attachments, others commenting on their ability to recognise individuals, and generally how the well-being of their animals affected them personally. Much discussion has centred on the farmer-animal relationships as being utilitarian and exploitative in nature given that most farm animals are sent to slaughter at some point (Lund & Olsson 2006). However, there is little doubt that caring about animals is central to farmers’ identity and culture (Tovey 2003; Porcher 2006) and an integral characteristic of being a ‘good farmer’ (De Greef *et al.* 2006; Dockes & Kling-Eveillard 2006; Lassen *et al.* 2006; Bruckmeier & Prutzer 2007). From the perspective of members of the public, the way that farmers in this study described their relationships with individual cows paints a compelling picture of dairy farmers as caring individuals who value their animals beyond just utilitarian value to humans. Our companion paper on views of members of the public (Spooner *et al.* 2014b) clearly demonstrated the importance of seeing animals as having intrinsic value and deserving of respect and the importance of personal relationships. In contrast to beef and pig farmers (Spooner *et al.* 2012, 2014b), the degree to which dairy farmers had relationships with individuals animals is quite different.

The majority of our participants expressed an emotional attachment with their animals that was often described as a consequence of the day-to-day interaction and long-term history that they had with individuals in the herd and their descendants. However, it was also clearly conveyed by the farmers that at some point the cows had to be sold but this could be emotionally taxing. As initially outlined by Lund and Olsson (2006), the selling of the animal becomes acceptable when there is an emotional attachment between the farmers and their animals that supports the animals having a ‘good life’ prior to their death (see also Singer 1975).

With regard to the need to sell their animals, some participants conveyed annoyance with the public who they believed thought they did so without emotion. For instance, one farmer stated that: “*People don’t think that you have a relationship and that it’s easy… just a business, get rid of the cows. There are people like that, just like there are people who treat their neighbours badly, right? But, for the most part, it is a business, you do have to make a living, so you can’t be too soft, but on the other hand, it’s incorrect to imagine that a farmer has no emotional attachment to his animals, because they’re living beings, they live and breathe with you, you work with them all day long”* (258).

This openly emotional attachment to their cows was conveyed by another farmer when describing his feelings after having to sell his two favourite cows: *“Personally speaking, I love my cows. And this may sound a bit bizarre, but if two of my favourite cows are leaving on the beef truck, I make sure that morning I have something else to do. I don’t like it. I really don’t like it”* (266). Similarly, in the words of another farmer, “….*When I first started it was a very emotional thing to depart from your animals… You know when I actually sat down beside an animal and bawled my eyes out because you know it really hurt”* (228).

Farmers also spoke of how their own feelings were affected by the cow’s state of well-being. For instance, one farmer said, *“When the cows are happy, I’m happy too. When I have a sick cow, I’m sick too, sometimes… my wife said always at night, ‘Put it on the side of the bed, all the trouble from the cows,’ otherwise it goes with you and you wake up, you think of that cow right away”* (194). Similarly, another farmer recounted the pleasure of seeing his animals content: *“So you come in the barn at 11 o’clock and check your cows out, because we do a late-night check, and they’re all laying in the stalls, they’re chewing their cud, and you know, the steam’s rising off, and they’re just happy. That’s a happy barn, and no noise, they’re all chewing their cud. That’s what makes me happy because I know my cows are happy”* (272).

Farmers also often made reference to individual cows within their herd. *“You know, some of our cows have been with us twelve years and stuff. That’s a long time… So, you can go three or four generations of animals and you remember – like, I remember some cows that we got rid of five, six years ago. I remember if… there was something about her, she had personality”* (170). One dairy farmer also recounted a ritual of petting one particular cow: “*We’ve got one cow right now, whenever we bring her to milk her, she’ll just stand there and wait, you just got to pet her on the head a couple of times… and once you pat her on the head a few times, she’s fine. Off she goes again”* (166). However, others felt that the ability to recognise individuals was more challenging on larger farms. For instance, one farmer suggested that on bigger farms one ended up managing groups of cows rather than individuals: *“…3,500 cows… you can’t get to know those 3,500 cows”* (256). The view that larger farms are not conducive to getting to know and caring for your animals is also echoed by members of the public (Spooner *et al.* 2014b).

Some of the participants also believed that the special relationship that they had with some of their animals was reciprocated. For instance, one couple spoke about how cows interacted differently with each of them: “*There’s cows that know* [me] *and anybody else that goes to milk them they’d milk different. There’s another old cow that you’ve never gotten along with, I get along with fine. It’s just they get to know you…. There’s one little cow that just hates me”* (234).

Farmers had different views about the treatment of cows on big farms, but not necessarily from first-hand experience. The issue of farm size and animal welfare is complex. Robbins *et al.* (2016) found little evidence of any relationship between animal welfare and farm size but did suggest that larger farms likely had increased opportunities to improve animal welfare given improved access to more resources or specialists. In contrast, small farms may have fewer challenges when trying to incorporate pasture access compared to big farms (Robbins *et al.* 2016). In the current study farmers felt that treatment of cows by handlers was equivalent on small and large farms but they did acknowledge that small farms may be better positioned to create calm environments for the animals: “*Our cattle, you can scrape out the barn or move around them and they just move out of the way, because they kind of trust it, because we don’t have many animals. But, when you’re on a larger scale, they tend to, I wouldn’t say they’re frightened, they’re just not secure”* (258). Other work has also reported differences in farmer perception of animal welfare in relation to farm size. For example, Australian sheep farmers perceived animal welfare to decline as farm size increases (Kılıç & Bozkurt 2013).

### Limitations

A limitation of this study is the challenge of having a discussion with farmers about the abstract concepts of animal welfare. For farmers whose daily work involves interacting with a variety of systems, animal welfare is one of a complex variety of facets. Also, this study was based on a small number of interviews and was not intended to be generalisable in the positivist, quantitative tradition (Carminati 2018). As such, we do not make claims regarding a larger population. However, the sample reflected the range in types of dairy production systems in Canada (although we had only two tie-stall farms) from locations across Canada and had similar average herd sizes. In addition, we believe that the results were supported by existing larger and more demographically balanced literature such that they provide valuable insights into the growing conversation about the future of the dairy industry. Finally, the study was conducted a number of years ago and to address this gap we have presented the findings in relation to current literature to provide insight into where things have or have not changed and why.

### Animal welfare implications

This study provides evidence that for the interviewed dairy farmers, being a dairy farmer is more than simply milking cows and making a profit. The data from this study revealed nuanced and deep-rooted beliefs associated with farmers generally accepting that they held a duty to provide good care to their animals. Farmers in this study valued a lifestyle that allowed them to have autonomy, to be hard working, and to work with animals. In terms of animal welfare, the majority of farmers emphasised the importance of the biological functioning, however, there was recognition that their animals *–* at least to some degree *–* lived emotional lives and it impacted farmers emotionally when their animals were not feeling well. When farmers spoke about the relevance of naturalness to animal welfare, they mostly spoke about access to pasture but there were differing views about the benefits and necessity. While comments mostly revolved around whether pasture was good or bad for health and good or bad for milk production, there was support for pasture contributing to positive welfare. Access to pasture along with lameness and cow-calf separation remain challenging welfare issues for the industry. While farmers valued the role that consumers or the public play in welfare, many farmers were frustrated regarding the public’s unrealistic expectations and the criticism that they were only in the business for profit. Similarly, there were few comments suggesting a willingness to change practices. However, in terms of the overall picture, this study also identified potential shared values with members of the public. For example, opportunities for natural living and for agency, attentiveness to individual animals, and the value of life over death. Finally, the emotional relationship that farmers developed with their animals highlights the values dairy farmers have for their animals beyond simply a utilitarian function. Overall, these shared values could contribute to constructive dialogue.

## References

[r1] Agriculture and Agri-Food Canada 2021 *Canadian Dairy Information Centre.* https://agriculture.canada.ca/en/sector/animal-industry/canadian-dairy-information-centre

[r2] Alonso ME, González-Montaña JR and Lomillos JM 2020 Consumers’ concerns and perceptions of farm animal welfare. Animals (Basel) 10: 385. 10.3390/ani10030385 32120935 PMC7143148

[r3] Atkinson D, von Keyserlingk MAG and Weary DM 2017 Benchmarking passive transfer of immunity and growth in dairy calves. Journal of Dairy Science 100: 3773–3782. 10.3168/jds.2016-11800 28237586

[r4] Balzani A and Hanlon A 2020 Factors that influence farmers’ views on farm animal welfare: A semi-systematic review and thematic analysis. Animals (Basel) 10(9): 1524. 10.3390/ani10091524 32872206 PMC7552314

[r5] Barkema HW, von Keyserlingk MAG, Kastelic JP, Lam, TJG, Luby MC, Roy J-P, LeBlanc SJ, Keefe GP and Kelton DF 2015 Invited review: Changes in the dairy industry affecting dairy cattle health and welfare. Journal of Dairy Science 98: 7426–7445. 10.3168/jds.2015-9377 26342982

[r6] Beaver A, Meagher RK, von Keyserlingk MAG and Weary DM 2019 Invited review: A systematic review of the effects of early separation on dairy cow and calf health. Journal of Dairy Science 102: 5784–5810. 10.3168/jds.2018-15603 31079908 PMC7094284

[r7] Bock BB, Van Huik MM, Prutzer M, Eveillard FK and Docke A 2007 Farmers’ relationship with different animals: the importance of getting close to the animals. Case studies of French, Swedish and Dutch cattle, pig and poultry farmers. International Journal of Sociology of Agriculture and Food 15(3): 108–125. 10.48416/ijsaf.v15i3.290

[r8] Bolton SE and von Keyserlingk MAG 2021 The dispensable surplus dairy calf: Is this issue a “Wicked Problem” and where do we go from here? Frontiers in Veterinary Science 8: 660934. 10.3389/fvets.2021.660934 33937380 PMC8079806

[r9] Bos JM, Bovenkerk B, Feindt PH, and van Dam YK 2018 The quantified animal: Precision livestock farming and the ethical implications of objectification. Food Ethics 2: 77–92. 10.1007/s41055-018-00029

[r10] Bran JA, Daros RR, von Keyserlingk MAG, LeBlanc SL and Hötzel MJ 2018 Cow-and herd-level factors associated with lameness in small-scale grazing dairy herds in Brazil. Preventative Veterinary Medicine 151: 79–86. 10.1016/j.prevetmed.2018.01.006 29496110

[r11] Breuer K, Hemsworth PH, Barnett JL, Matthews LR and Coleman GJ 2000 Behavioural response to humans and the productivity of commercial dairy cows. Applied Animal Behaviour. Science 66: 273–288. 10.1016/S0168-1591(99)00097-0 10700627

[r12] Bruckmeier K and Prutzer M 2007 Swedish pig farmers and their perspectives on animal welfare – a case study. British Food Journal 109(11): 906–918. 10.1108/00070700710835714

[r13] Bruni D, Perconti P and Plebe A 2018 Anti-anthropomorphism and its limits. Frontiers in Psychology 9: 2205. 10.3389/fpsyg.2018.02205 30498465 PMC6249301

[r14] Burton RJF, Peoples S and Cooper MH 2012 Building ‘cowshed cultures’: A cultural perspective on the promotion of stockmanship and animal welfare on dairy farms. Journal of Rural Studies 28: 174–187. 10.1016/j.jrurstud.2011.12.003

[r15] Butler D and Holloway L 2016 Technology and restructuring the social field of dairy farming: Hybrid capitals, ‘stockmanship’ and automatic milking systems. Sociologia Ruralis 56: 513–530. 10.1111/soru.12103

[r16] Cardoso CS, Hötzel MJ, Weary DM, Robbins JA and von Keyserlingk MAG 2016 Imagining the ideal dairy farm. Journal of Dairy Science 99: 1663–1671. 10.3168/jds.2015-9925 26709190

[r17] Carminati L 2018 Generalizability in qualitative research: A tale of two traditions. Qualitative Health Research 28: 2094–2101. 10.1177/1049732318788379 30043686

[r18] Chapinal N, Weary DM, Collings L and von Keyserlingk MAG 2014 Lameness and hock injuries improve on farms participating in an assessment program. Veterinary Journal 202: 646–648. 10.1016/j.tvjl.2014.09.018 25447801

[r19] Charlton GL and Rutter SM 2017 The behaviour of housed dairy cattle with and without pasture access: A review. Applied Animal Behaviour Science 192: 2–9. 10.1016/j.applanim.2017.05.015

[r20] Clark B, Stewart GB, Panzone LA, Kyriazakis I and Frewer LJ 2016 A systematic review of public attitudes, perceptions and behaviours towards production diseases associated with farm animal welfare. Journal of Agricultural and Environmental Ethics 29: 455–478. 10.1007/s10806-016-9615

[r21] Coleman GJ, McGregor M, Hemsworth PH, Boyce J and Dowling S 2003 The relationship between beliefs, attitudes and observed behaviours of abattoir personnel in the pig industry. Applied Animal Behaviour Science 82: 189–200. 10.1016/S0168-1591(03)00057-1

[r22] Cook NB 2017 Assessment of cattle welfare: Common animal-based measures. In: Tucker C (ed) Advances in Cattle Welfare pp 27–53. Woodhead Publishing: UK. 10.1016/B978-0-08-100938-3.00002-4

[r23] Costa JHC, von Keyserlingk MAG and Weary DM 2016 Invited review: Effects of group housing of dairy calves on behavior, cognition, performance and health. Journal of Dairy Science 99: 2453–2467. 10.3168/jds.2015-10144 26874423

[r24] Dawson LC, Dewey CE, Stone EA, Guerin MT and Niel L 2016 A survey of animal welfare experts and practicing veterinarians to identify and explore key factors thought to influence canine and feline welfare in relation to veterinary care. Animal Welfare 25: 125–134. 10.7120/09627286.25.1.125

[r25] De Greef K, Staufleu F and de Lauwere C 2006 A simple value distinction approach aids transparency in farm animal welfare debate. Journal of Agricultural and Environmental Ethics 19: 57–66. 10.1007/s10806-005-4527-1

[r26] DFC–NFACC (Dairy Farmers of Canada) 2018 *ProAction–Leading the way for sustainable dairy farming.* https://www.dairyfarmers.ca/Media/Files/proaction/proaction_ang_lr15.pdf

[r27] Dockès AC and Kling-Eveillard F 2006 Farmers’ and advisers’ representations of animals and animal welfare. Livestock Science 103: 243–249. 10.1016/j.livsci.2020.104057

[r28] Dolby N and Litster A 2019 Animal welfare and animal rights. Society & Animals 27: 575–594. 10.1163/15685306-12341493

[r29] Duve LR and Jensen MB 2012 Social behavior of young dairy calves housed with limited or full social contact with a peer. Journal of Dairy Science 95: 5936–5945. 10.3168/jds.2012-5428 22901479

[r30] Espejo LA and Endres MI 2007 Herd-level risk factors for lameness in high-producing Holstein cows housed in freestall barns. Journal of Dairy Science 90: 306–314. 10.3168/jds.S0022-0302(07)72631-0 17183098

[r31] Festinger L 1962 Cognitive dissonance. Scientific American 207: 93–107. 10.1038/scientificamerican1062-93 13892642

[r32] Franks B and Higgins ET 2012 Effectiveness in human and other animals. In: Olson KM and Zanna MP (Eds.) Advances in Experimental Social Psychology, *Vol* 45 pp 285–346. Academic Press: New York, NY, USA. 10.1016/B978-0-12-394281-4.00006-4

[r33] Fraser D, Weary DM, Pajor EA and Milligan BN 1997 A scientific conception of animal welfare that reflects ethical concerns. Animal Welfare 6: 187–205.

[r34] Friedman G and Himmelstein J 2006 Resolving conflict together: The understanding-based model of mediation. Journal of Dispute Resolution 2: 1–32.

[r35] Gauly M, Bollwein H, Breves G, Brügemann K, Dänicke S, Daş G, Demeler J, Hansen H, Isselstein J, Konig S, Lohölter M, Martinsohn M, Meyer U, Potthoff M, Sanker C, Schröder B, Wrage N, Meibaum B, Von Samson-Himmelstjerna G, Stinshoff H and Wrenzycki C 2013 Future consequences and challenges for dairy cow production systems arising from climatechange in Central Europe - A review. Animal 7: 843–859. 10.1017/S1751731112002352 23253935

[r36] Glaser B and Strauss A 1967 The Discovery of Grounded Theory. Aldine Publishing Company: Hawthorne, NY, USA.

[r37] Hammersley M and Atkinson P 2007 Ethnography: Principles and Practice. Routledge: London, UK.

[r38] Hansson H and Lagerkvist CJ 2016 Dairy farmers’ use and non-use values in animal welfare: Determining the empirical content and structure with anchored best-worst scaling. Journal of Dairy Science 99: 579–592. 10.3168/jds.2015-9755 26547638

[r39] Heggem R 2014 Exclusion and inclusion of women in Norwegian agriculture: Exploring different outcomes of the ‘tractor gene.’ Journal of Rural Studies 34: 263–271. 10.1016/j.jrurstud.2014.03.002

[r40] Heminthavong K 2018 *Canada’s Supply Management System.* Government of Canada Publication No 2018-42-E. https://epe.lac-bac.gc.ca/100/201/301/weekly_acquisitions_list-ef/2019/19-03/publications.gc.ca/collections/collection_2019/bdp-lop/bp/YM32-2-2018-42-eng.pdf

[r41] Hemsworth PH, Coleman GJ, Barnett JL and Borg S 2000 Relationships between human-animal interactions and productivity of commercial dairy cows. Journal of Animal Science 78: 2821–2831. 10.2527/2000.78112821x 11063304

[r42] Hendricks J, Weary DM and von Keyserlingk MAG 2022 Veterinarian perceptions on the care of surplus dairy calves. Journal of Dairy Science 105: 6870–6879. 10.3168/jds.2022-22051 35787329

[r43] Hsieh HF, and Shannon SE 2005. Three approaches to qualitative content analysis. Qualitative Health Research 15: 1277–1288. 10.1177/1049732305276687 16204405

[r44] Jackson A, Green M, Millar K and Kaler J 2020 Is it just about grazing? UK citizens have diverse preferences for how dairy cows should be managed. Journal of Dairy Science 103: 3250–3263. 10.3168/jds.2019-17111 32057434

[r45] Jensen KC, Oehm AW, Campe A, Stock A, Woudstra S, Feist M, Müller KE, Hoedemaker M and Merle R 2022 German farmers’ awareness of lameness in their dairy herds. Frontiers in Veterinary Science 9: 866791. 10.3389/fvets.2022.866791 35400109 PMC8987770

[r46] Johnsen JF, Zipp KA, Kälber T, de Passillé AM, Knierim U, Barth K and Mejdell CM 2016 Is rearing calves with the dam a feasible option for dairy farms? — Current and future research. Applied Animal Behaviour Science 181: 1–11. 10.1016/j.applanim.2015.11.011

[r47] Kauppinen T, Vainio A, Valros A, Rita H and Vesala K 2010 Improving animal welfare: Qualitative and quantitative methodology in the study of farmers’ attitudes. Animal Welfare 19: 523–536.

[r48] Kiger ME and Varpio L 2020 Thematic analysis of qualitative data: AMEE Guide No. 131. Medical Teachear 42: 846–854. 10.1080/0142159X.2020.1755030 32356468

[r49] Kılıç I and Bozkurt Z 2013 The relationship between farmers’ perceptions and animal welfare standards in sheep farms. Asian-Australasian Journal of Animal Science 26: 1329–1338. 10.5713/ajas.2013.13124 PMC4093410 25049916

[r50] Lacetera N 2018 Impact of climate change on animal health and welfare. Animal Frontiers 9: 26–31. 10.1093/af/vfy030 32002236 PMC6951873

[r51] Lassen J, Sandøe P and Forkman B 2006 Happy pigs are dirty! — Conflicting perspectives on animal welfare. Livestock Science 103: 221–230. 10.1016/j.livsci.2006.05.008

[r52] Leach KA, Whay HR, Maggs CM, Barker ZE, Paul ES, Bell AK and Main DC 2010 Working towards a reduction in cattle lameness: 2. Understanding dairy farmers’ motivations. Research in Veterinary Science 89(2): 318–323. 10.1016/j.rvsc.2010.02.017 20413137

[r53] Lomb J, Mauger A, von Keyserlingk MAG and Weary DM 2021 Effects of positive reinforcement training for heifers on responses to a subcutaneous injection. Journal of Dairy Science 104: 6146–6158. 10.3168/jds.2020-19463 33685711

[r54] Lund V and Olsson IA 2006 Animal agriculture: symbiosis, culture, or ethical conflict? Journal of Agricultural and Environmental Ethics 19: 47–56. 10.1007/s10806-005-4378-9

[r55] Maher JW, Clarke A, Byrne AW, Doyle R, Blake M and Barrett D 2021 Exploring the opinions of Irish Dairy Farmers regarding male dairy calves. Frontiers in Veterinary Science 8: 635565. 10.3389/fvets.2021.635565 33959649 PMC8093389

[r56] McGuire J, Morton LW and Cast AD 2013 Reconstructing the good farmer identity: shifts in farmer identities and farm management practices to improve water quality. Agriculture and Human Values 30: 57–69. 10.1007/s10460-012-9381-y

[r57] Meijboom FLB and Stafleu FR 2016 Farming ethics in practice: from freedom to professional moral autonomy for farmers. Agriculture and Human Values 33: 403–414. 10.1007/s10460-015-9641-8

[r58] Muhammad M, Stokes JE, Morgans L and Manning L 2022 The social construction of narratives and arguments in animal welfare discourse and debate. Animals 12: 2582. 10.3390/ani12192582 36230322 PMC9559530

[r59] Mutua EK and Haskell MJ 2022 Factors contributing to milk yield variation among cows in a cow–calf contact system in early lactation. *Journal of Dairy Science* Communications 3(1): 55–58. 10.3168/jdsc.2021-0143 PMC9623780 36340674

[r60] National Farm Animal Care Council 2009 *Code of Practice for the Care and Handling of Dairy Cattle.* https://www.nfacc.ca/codes-of-practice/dairy-cattle

[r61] Neave HW, Sumner CL, Henwood RJT, Zobel G, Saunders K, Thoday H, Watson T and Webster JR 2022 Dairy farmers’ perspectives on providing cow-calf contact in the pasture-based systems of New Zealand. Journal of Dairy Science 105: 453–467. 10.3168/jds.2021-21047 34696913

[r62] Niska M, Vesala HT and Vesala KM 2012 Peasantry and entrepreneurship as frames for farming: reflections on farmers’ values and agricultural policy discourses. Sociologia Ruralis 52: 453–468. 10.1111/j.1467-9523.2012.00572.x

[r63] NMPF (National Milk Farmers Federation) 2020 *Farmers Assuring Responsible Management (FARM): Animal Care Reference Manual.* https://nationaldairyfarm.com/wp-content/uploads/2018/10/Version-3-Manual-1.pdf

[r64] Olmos G, Bran JA, von Keyserlingk AMG and Hötzel MJ 2018 Lameness on Brazilian pasture-based dairies – Part 2: Conversations with farmers and dairy consultants. Preventive Veterinary Medicine 157: 115–124. 10.1016/j.prevetmed.2018.06.009 30086839

[r65] Paquet PC and Darimont CT 2010 Wildlife conservation and animal welfare: two sides of the same coin? Animal Welfare 19: 177–190. 10.1017/S0962728600001433

[r66] Phillips CJC, Wojciechowska J, Meng J and Cross N 2009 Perceptions of the importance of different welfare issues in livestock production. Animal 3: 1152–1166. 10.1017/S1751731109004479 22444845

[r67] Placzek M, Christoph-Schulz I and Barth K 2021 Public attitude towards cow-calf separation and other common practices of calf rearing in dairy farming—A review. Organic Agriculture 11: 41–50. 10.1007/s13165-020-00321-3

[r68] Porcher J 2006 Well-being and suffering in livestock farming: living conditions at work for people and animals. Sociologie du Travail 48(1): e56–e70. 10.1016/j.soctra.2006.02.001

[r69] Ritter C, Mills KE, Weary DM and von Keyserlingk MAG 2020 Perspectives of western Canadian dairy farmers on the future of farming. Journal of Dairy Science 103: 10273–10282. 10.3168/jds.2020-18430 32952024

[r70] Robbins JA, von Keyserlingk MAG, Fraser D and Weary DM 2016 Invited review: Farm size and animal welfare. Journal of Animal Science 94: 5439–5455. 10.2527/jas.2016-0805 28046157

[r71] Robbins JA, Weary DM, Schuppli CA and von Keyserlingk MAG 2015 Stakeholder views on treating pain due to dehorning dairy calves. Animal Welfare 24: 399–406. 10.7120/09627286.24.4.399

[r72] Rollin BE 2011 Animal rights as a mainstream phenomenon. Animals 1:102–115. 10.3390/ani1010102 26486217 PMC4552208

[r73] Rutledge PB 2009 *Common Ground in the Arbitration Debate*: *Yearbook on Arbitration and Mediation.* https://ssrn.com/abstract=1392465

[r74] Schillings J, Bennett R and Rose DC 2021 Exploring the potential of precision livestock farming technologies to help address farm animal welfare. Frontiers in Animal Science 2: 639678. 10.3389/fanim.2021.639678

[r75] Shields S, Shapiro P and Rowan A 2017 Decade of progress toward ending the intensive confinement of farm animals in the United States. Animals 7(5): 40. 10.3390/ani7050040 28505141 PMC5447922

[r76] Silva S, Magalhaes-Sant’Ana M, Borlido Santos J and Olsson IAS 2013 Comfort, health and production: Portuguese dairy farmers talk about animal welfare. In: Rocklinsberg H and Sandin P (Eds.) The Ethics of Consumption: The Citizen, the Market and the Law pp 209–214. Eursafe: Uppsala, Sweden.

[r77] Singer P 1975 Animal Liberation: A New Ethics for Our Treatment of Animals. Harper Collins: New York, NY, USA.

[r78] Sirovica L V Hendricks J Ritter C Weary DM Gulati S and von Keyserlingk MAG 2022 Public perceptions of dairy cow-calf management systems differing in type of social and maternal contact. Journal of Dairy Science 105: 3248–3268. 10.3168/jds.2021-21344 35094864

[r79] Skarstad G, Terragni L and Torjusen H 2007 Animal welfare according to Norwegian consumers and farmers: Definitions and implications. International Journal of Sociology and Agriculture and Food 15(3). 10.48416/ijsaf.v15i3.285

[r80] Smid AMC, Inberg PHJ, de Jong S, Sinclair S, von Keyserlingk MAG, Weary DM and Barkema HW 2022 Perspectives of Western Canadian dairy farmers on providing outdoor access for dairy cows. Journal of Dairy Science 104: 10158–10170. 10.3168/jds.2021-20342 34218920

[r81] Smid AMC, Weary DM and von Keyserlingk MAG 2020 The influence of different types of outdoor access on dairy cattle behavior. Frontiers in Veterinary Science 7: 257. 10.3389/fvets.2020.00257 32478110 PMC7238891

[r82] Špinka M 2019 Animal agency, animal awareness and animal welfare. Animal Welfare 28(1): 11–20. 10.7120/09627286.28.1.011

[r83] Spooner JM, Schuppli CA and Fraser D 2012 Attitudes of Canadian beef farmers toward animal welfare. Animal Welfare 21: 273–283. 10.7120/09627286.21.2.273

[r84] Spooner JM, Schuppli CA and Fraser D 2014a Attitudes of Canadian pig farmers toward animal welfare. Journal of Agricultural and Environmental Ethics 27: 569–589. 10.1007/s10806-013-9477-4

[r85] Spooner JM, Schuppli CA and Fraser D 2014b Attitudes of Canadian citizens toward farm animal welfare: A qualitative study. Livestock Science 163: 150–158. 10.1016/j.livsci.2014.02.011

[r86] Stock PV and Forney J 2014 Farmer autonomy and the farming self. Journal of Rural Studies 36: 160–171. 10.1016/j.jrurstud.2014.07.004

[r87] Sumner CL and von Keyserlingk MAG 2018 Canadian dairy cattle veterinarian perspectives on calf welfare. Journal of Dairy Science 101: 10303–10316. 10.3168/jds.2018-14859 30197138

[r88] Sumner CL, von Keyserlingk MAG and Weary DM 2018 How benchmarking motivates farmers to improve dairy calf management. Journal of Dairy Science 101: 3323–3333. 10.3168/jds.2017-13596 29397181

[r89] Sutherland LA and Darnhofer I 2012 Of organic farmers and ‘good farmers’: changing habitus in rural England. Journal of Rural Studies 28: 232–240. 10.1016/j.jrurstud.2012.03.003

[r90] Te Velde H, Aarts N and van Woerkum C 2002 Dealing with ambivalence: Farmers’ and consumers’perceptions of animal welfare in livestock breeding. Journal of Agricultural and Environmental Ethics 15: 203–219. 10.1023/A:1015012403331

[r91] Tovey H 2003 Theorising nature and society in sociology: the invisibility of animals. Sociologia Ruralis 43: 196–213. 10.1177/1368431016681305

[r92] van Gelderen M and Jansen P 2006 Autonomy as a start-up motive. Journal of Small Business and Enterprise Development 13: 23–32. 10.1108/14626000610645289

[r93] Vanhonacker F, Verbeke W, Van Poucke E and Tuyttens FA 2007 Segmentation based on consumers’ perceived importance and attitude toward farm animal welfare. International Journal of Sociology and Food Agriculture 15(3): 91–107. 10.48416/ijsaf.v15i3.286

[r94] Vanhonacker F, Verbeke W, Van Poucke E and Tuyttens FA 2008 Do citizens and farmers interpret the concept of farm animal welfare differently? Livestock Science 116: 126–136. 10.1016/j.livsci.2007.09.017

[r95] Ventura BA, von Keyserlingk MAG, Schuppli CA and Weary DM 2013 Views on contentious practices in dairy farming: The case of early cow-calf separation. Journal of Dairy Science 96: 6105–6116. 10.3168/jds.2012-6040 23791487

[r96] Ventura BA, Weary DM, Giovanetti AS and von Keyserlingk MAG 2016 Veterinary perspectives on cattle welfare challenges and solutions. Livestock Science 193: 95–102. https://doi.org/10.1016/j.livsci.2016.10.004

[r97] Verbeke W 2009 Stakeholder, citizen and consumer interests in farm animal welfare. Animal Welfare 18: 325–333.

[r98] von Keyserlingk MAG, Cestari AA, Franks B, Fregonesi JA and Weary DM 2017 Dairy cows value access to pasture as highly as fresh feed. Scientific Reports 7: 44953. 10.1038/srep44953 28332567 PMC5362966

[r99] Weary DM and von Keyserlingk MAG 2017 Invited review: Public concerns about dairy cow welfare — how should the industry respond? Animal Production Science 57: 1201–1209. 10.1071/AN16680

[r100] Werkheiser I 2018 Precision livestock farming and farmers’ duties to livestock. Journal of Agriculture and Environmental Ethics 31(2): 181–195. 10.1007/s10806-018-9720-0

[r101] Wikman I, Hokkanen A-H, Pastell M, Kauppinen T, Valros A and Hänninen L 2013 Dairy farmer attitudes to pain in cattle in relation to disbudding calves. Journal of Dairy Science 96: 6894–6903. 10.3168/jds.2012-6128 24054284

[r102] Winder CB, Bauman CA, Duffield TF, Barkema HW, Keefe GP, Dubuc J, Uehlinger F and Kelton DF 2018 Canadian National Dairy Study: Heifer calf management. Journal of Dairy Science 101: 10565–10579. 10.3168/jds.2018-14680 30172400

[r103] Wynands EM, Roche SM, Cramer G, Ventura BA 2021 Dairy farmer, hoof trimmer, and veterinarian perceptions of barriers and roles in lameness management. Journal of Dairy Science 104: 11889–11903. 10.3168/jds.2021-20603 34454749

[r104] Yeates JW 2017 How good? Ethical criteria for a ‘good life’ for farm animals. Journal of Agricultural and Environmental Ethics 30: 23–35. 10.1007/s10806-017-9650-2

